# YTHDF1 Negatively Regulates *Treponema pallidum*-Induced Inflammation in THP-1 Macrophages by Promoting SOCS3 Translation in an m6A-Dependent Manner

**DOI:** 10.3389/fimmu.2022.857727

**Published:** 2022-04-04

**Authors:** Zhijia Li, Muzhou Teng, Yinbo Jiang, Litian Zhang, Xi Luo, Yuhui Liao, Bin Yang

**Affiliations:** Dermatology Hospital, Southern Medical University, Guangzhou, China

**Keywords:** syphilis, *Treponema pallidum*, inflammation, m6A methylation, macrophage

## Abstract

**Background:**

Previous studies have confirmed that the bacterium *Treponema pallidum* (TP) or its proteins provide signals to macrophages that induce an inflammatory response; however, little is known about the negative regulation of this macrophage-mediated inflammatory response during syphilis infection or the underlying mechanism. Recent evidence suggests the role of the RNA modification, N^6^-adenosine methylation (m6A), in regulating the inflammatory response and pathogen-host cell interactions. Therefore, we hypothesized that m6A plays a role in the regulation of the inflammatory response in macrophages exposed to TP.

**Methods:**

We first assessed m6A levels in TP-infected macrophages differentiated from the human monocyte cell line THP-1. The binding and interaction between the m6A “writer” methyltransferase-like 3 (METTL3) or the m6A “reader” YT521-B homology (YTH) domain-containing protein YTHDF1 and the suppressor of cytokine signaling 3 (SOCS3), as a major regulator of the inflammatory response, were explored in differentiated TP-infected THP-1 cells as well as in secondary syphilitic lesions from patients. The mechanisms by which YTHDF1 and SOCS3 regulate the inflammatory response in macrophages were assessed.

**Results and Conclusion:**

After macrophages were stimulated by TP, YTHDF1 was upregulated in the cells. YTHDF1 was also upregulated in the syphilitic lesions compared to adjacent tissue in patients. YTHDF1 recognizes and binds to the m6A methylation site of *SOCS3* mRNA, consequently promoting its translation, thereby inhibiting the JAK2/STAT3 pathway, and reducing the secretion of inflammatory factors, which results in anti-inflammatory regulation. This study provides the first demonstration of the role of m6A methylation in the pathological process of syphilis and further offers new insight into the pathogenesis of TP infection.

## Introduction

Syphilis is a sexually transmitted disease that is caused by infection with the bacterium *Treponema pallidum* (TP). The annual incidence of syphilis has increased in recent years, and its prevalence has caused serious public health problems. TP can invade the skin, mucosal membranes, and nerves, among other sites, thereby causing multi-system damage (1). As an important subpopulation of cells that are involved in the innate immune response, macrophages bind to TP lipoprotein *via* the pattern recognition receptor on the macrophage cell surface, which leads to their self-activation and initiation of the immune response (2, 3). Macrophages eradicate TP directly through phagocytosis and the secretion of a large number of inflammatory cytokines (4). In addition, the inflammatory cytokines secreted by macrophages can further stimulate CD4+ T cells to produce Th1-type cytokines, such as interferon (IFN)-γ and tumor necrosis factor (TNF)-α, to indirectly eliminate TP (4), which also occurs in syphilitic lesions (5, 6). As these TP infection-induced immune responses can cause tissue damage, macrophages may also negatively regulate the inflammatory response upon TP stimulation through related pathways to prevent overwhelming systemic inflammation. Our previous study revealed that TP infection upregulated the expression of the microRNA miR-101-3p, which inhibited the Toll-like receptor 2 signaling pathway and reduced cytokine production (7). However, few studies have focused on the negative regulation of the inflammatory response in macrophages during syphilis infection, and the underlying mechanism remains to be clarified.

N6-adenosine methylation (m6A) is the most abundant mRNA modification, which plays an important role in the regulation of various pathophysiological processes, including the inflammatory response and immune regulation, and has thus attracted widespread attention in recent years (8, 9). m6A methylation is dynamically regulated by three regulatory factors—writers, erasers, and readers. m6A methylation participates several mRNA metabolic processes, including mRNA degradation, splicing, folding, nucleation, and translation (10). Writers, methyltransferase-like (METTL) 3 (METTL3), METTL14, and other methyltransferases form a protein complex that modifies mRNAs *via* m6A methylation in the nucleus. Moreover, demethylases (erasers), such as FTO and ALKBH5, can oxidize and remove some methyl sites (11, 12). Subsequently, the readers, including members of the YT521-B homology (YTH) domain-containing protein family (YTHDF1/2/3 and YTHDC1/2) and heterogeneous nuclear ribonucleoprotein (HNRNP) proteins, can recognize and interact with m6A sites (11, 12). For example, YTHDF1 controls mRNA degradation and promotes translation efficiency (10).

Given the importance of m6A methylation in the regulation of genes that are involved in immune and inflammatory responses, we hypothesized that this modification might also be involved in the inflammatory response of macrophages during syphilis infection. To test this hypothesis, we compared the levels of m6A methylation in macrophages cultured *in vitro* with and without TP infection, and the influence of TP infection on the changes in m6A-related proteins. We further investigated the effect of TP infection in macrophages using knockdown and overexpression experiments of m6A-related proteins and examined the changes in the gene expression profiles and impact of SOCS3 expression. SOCS3 is a negative regulator of the JAK2-STAT3 pathway, which plays an important role in inflammation and the response to infection.

## Materials and Methods

### Cell Culture and Infection

THP-1 cells, a human monocyte cell line, were provided by Dr. Chuncai Gu, PhD (Nanfang Hospital, Southern Medical University, Guangzhou, China). THP-1 cells were grown in culture media, containing RPMI 1640 media (HyClone, Logan, UT, USA) supplemented with 10% fetal bovine serum (Gibco, Australia) and 1% penicillin-streptomycin. Cells were grown in a humidified 5% CO_2_ atmosphere at 37°C. The culture media was then supplemented with 100 ng/mL phorbol 12-myristate 13-acetate (Sigma-Aldrich, St. Louis, MO, USA) and incubated for 24 h to induce the differentiation of the monocytes into M0 macrophages. The obtained macrophages were then cultured in a media without penicillin-streptomycin. The cells were split into two treatment groups cultured with TP (Nichols strain, kindly provided by Yinbo Jiang, Dermatology Hospital, Southern Medical University, Guangzhou, China) at a multiplicity of infection (MOI) of 20:1 (TP:cell) or phosphate-buffered saline (PBS) as the control group. The two treatment groups were incubated for 24 h.

### Small Interfering RNA (siRNA)-Mediated Knockdown and Plasmid Transfection

*YTHDF1, METTL3, SOCS3*, and control siRNAs (Sangon Biotech, Shanghai, China) were transfected into THP-1 cells at a final concentration of 10 nM using RNA MAX siRNA Transfection Reagent (Invitrogen) according to the manufacturer’s instructions. THP-1 cells were incubated at 37°C in a CO_2_ incubator for 48 h, and the transfection efficiency was evaluated using western blotting analysis as described below. The siRNA sequences are listed in **Supplementary Table S1**.

THP-1 cells were transfected with the SOCS3-expressing plasmid (Sangon Biotech, Shanghai, China) using Lipo3000 (Invitrogen).

### Immunofluorescence

The Ethical Approval Board of Dermatology Hospital, Southern Medical University, China approved the use of tissue wax blocks from patients with secondary syphilis (GDDHLS-20180510). For immunofluorescence analysis of the tissues, the sections were permeabilized with 0.25% Triton X-100 for 0.5 h and blocked with 5% goat serum for 1 h. Tissue sections were then incubated with primary antibodies against CD68 (Cat. No. 66231-2-Ig, Proteintech, Wuhan, Hubei, China) and YTHDF1 (Cat. No. 17479-1-AP, Proteintech) at 4°C overnight, followed by incubation with the secondary antibody, Alexa Fluor^®^ 488 donkey anti-rabbit IgG (H+L) (A21206, Life Technologies) or Alexa Fluor^®^ 594 donkey anti-mouse IgG (H+L) (A21203, Life Technologies). The coverslips were mounted onto slides using an antifade mounting medium containing DAPI. The images were captured using a fluorescence microscope.

### Enzyme-Linked Immunosorbent Assay (ELISA)

Levels of cytokines (TNF-α and IL-β) in the supernatant of the THP-1 cells were determined using ELISA kits (DAKEWE, Beijing, China) according to the manufacturer’s instructions.

### Reverse Transcription-Quantitative Polymerase Chain Reaction (RT-qPCR)

RT-qPCR was performed to determine *SOCS3* mRNA levels. Total RNA was extracted from THP-1 cells using TRIzol reagent (Life Technologies), following the manufacturer’s protocol. cDNA was synthesized using a PrimeScript RT Reagent Kit (Takara, Japan). The cDNA was then used as a template for qPCR with SYBR Green Master Mix (Takara, Japan), according to the manufacturer’s protocol. After amplification, cycle threshold (Ct) and ΔΔCt values were obtained for quantification.

### Western Blotting

Total protein from each group of cells was isolated and measured using a BCA Protein Assay Kit (EpiZyme, Shanghai). Total proteins were separated using sodium dodecyl sulfate-polyacrylamide gel electrophoresis and transferred to polyvinylidene fluoride membranes. The membrane was blocked with Protein-Free Rapid Blocking Buffer (EpiZyme, Shanghai) for 15 min at room temperature and incubated with the following primary antibodies overnight at 4°C: anti-METTL3 (Cat. No. 15073-1-AP, Proteintech), anti-METTL14 (Cat. No. 26158-1-AP, Proteintech), anti-YTHDF1 (Cat. No. 17479-1-AP, Proteintech), anti-SOCS3 (Cat. No. 14025-1-AP, Proteintech), anti-JAK2 (17670-1-AP, Proteintech), anti-p-JAK2 (ab32101, Abcam), anti-STAT3 (Cat. no. 60199-1-Ig, Proteintech), anti-p-STAT3 (ab267373; Abcam), and anti-GAPDH (Cat. No.60004-1-Ig, Proteintech), as a loading control. After washing thrice, the PVDF membrane was incubated with the secondary antibody for 2 h at room temperature. The membranes were visualized using chemiluminescence. The signal intensities were quantified using the ImageJ software (version 1.49).

### Flow Cytometry

Single-cell suspensions were prepared from cultured THP-1 cells and incubated with anti-CD206 (374208) and anti-CD86 (321110) antibodies (both from Abcam) for 30 min at 4°C for cell-surface staining. Data were recorded on a BD Celesta flow cytometer and analyzed using the FlowJo software (V10, Treestar).

### m6A Quantitation

The m6A levels were measured using an m6A RNA Methylation Assay Kit (Colorimetric) (Abcam, No. ab185912) according to the manufacturer’s instructions.

### RNA-Sequencing

Total whole-cell RNA from the control and TP-infected cells was extracted using the TRIzol reagent (Invitrogen) and quantified using a Qubit^®^ RNA Assay Kit on a Qubit^®^ 2.0 fluorometer (Life Technologies, CA, USA). The RNA purity was assessed using a NanoPhotometer spectrophotometer (IMPLEN, CA, USA). The cDNA library was constructed using NEBNext^®^ UltraTM RNA Library Prep Kit for Illumina^®^ (NEB, USA). Sequencing was performed using an Illumina NovaSeq 6000 platform (Novogene, Beijing, China). High-quality reads were mapped to the human reference genome (hg38) using the HISAT2 program. Differential expression analysis of the two groups was performed using the DESeq2 package in R (1.10.1). Genes with expression levels differing between groups at an adjusted P value <0.05 were considered to be significantly differentially expressed. Sequencing was performed in three independent biological replicates.

### m6A RNA Immunoprecipitation (MeRIP)-qPCR

Total RNA was extracted using TRIzol reagent (*Invitro*gen) and fragmented using a fragmentation buffer. The fragmented mRNA (100 ng) was then used as an input control, and the remainder of the RNA was incubated with magnetic ChIP protein A/G magnetic beads to isolate methylated RNA according to the manufacturer’s instructions (Magna MeRIP m6A Assay, Millipore, 17-10499). MeRIPed RNA was analyzed using RT-qPCR.

### RIP-qPCR

The procedure for RIP-qPCR was adapted from a previous study (13). THP-1 cells transfected with siNC, siMETTL3, or siYTHDF1 were washed twice with PBS, collected, and resuspended in immunoprecipitation lysis buffer. 10% of the cell lysate were saved as input, and the remaining sample was incubated with IgG antibody, YTHDF1 antibody, and protein G beads (Invitrogen) overnight at 4°C. After washing three times with wash buffer, the co-precipitated RNAs were extracted using TRIzol reagent and ethanol-precipitation with glycogen. Fold enrichment was determined by RT-qPCR.

### Statistical Analysis

Data were expressed as the mean ± standard deviation, and statistical significance was determined using an unpaired Student *t*-test for the two groups and ANOVA for multiple groups with SPSS 19.0 and GraphPad Prism 5.0. Statistical significance was set at P < 0.05.

## Results

### Effect of TP on m6A Methylation in Macrophages *In Vitro* and Secondary Syphilitic Lesions

**Figure 1:**
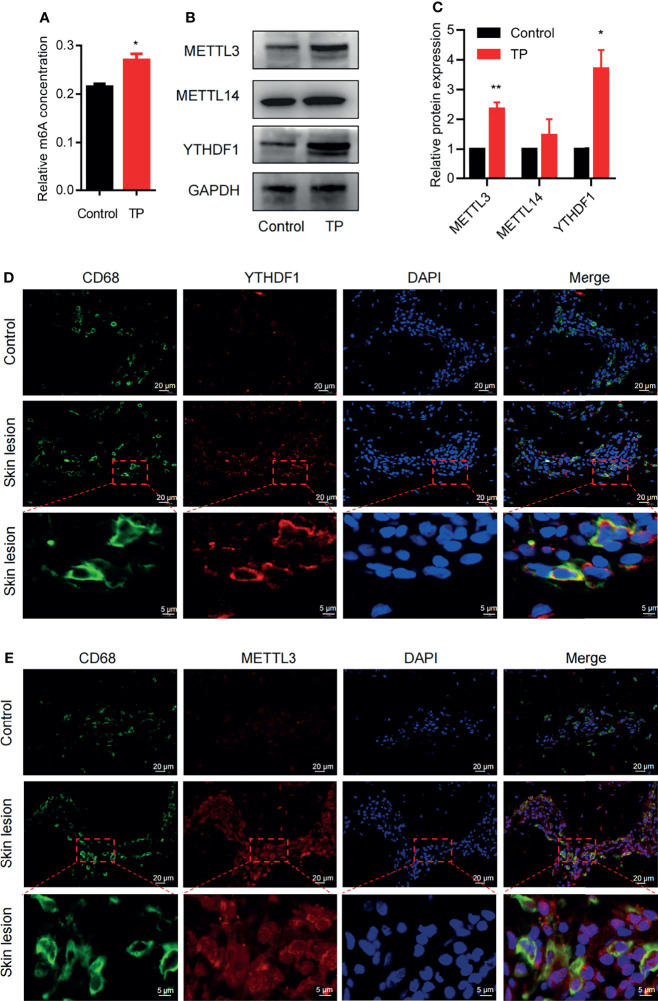
m6A methylation: the m6A writer METTL3 and the reader YTHDF1 are upregulated in TP-infected macrophages. **(A)** Colorimetric quantification of m6A methylation in RNA from THP-1 cells with or without TP infection. **(B, C)** Western blot analysis of METTL3, METTL14, and YTHDF1 in THP-1 cells with or without TP infection. GAPDH was used as a control. *P < 0.05, **P < 0.01. **(D, E)** Immunofluorescence analysis of YTHDF1 or METTL3 in CD68+ macrophages in secondary syphilitic lesions and paired non-lesional skin tissue. Scale bar: 20 μm or 5 μm.

To explore whether m6A methylation is related to the inflammatory response of macrophages in syphilis infection, we first investigated m6A levels in TP-infected macrophages. Compared to the control, TP stimulation upregulated the m6A levels (**Figure 1A**). We also investigated the expression patterns of the m6A writers (METTL3 and METTL14) and readers (YTHDF1) in TP-infected macrophages. After stimulation with TP for 24 h (MOI = 20:1), the expression levels of METTL3 and YTHDF1 were significantly elevated compared with those in the control group (**Figures 1B, C** and **Supplementary Figure S1**). Similarly, immunofluorescence analysis showed upregulated expression of METTL3 and YTHDF1 in CD68+ macrophages from secondary syphilitic lesions compared with those of paired healthy skin tissue samples (**Figures 1D, E**). These data suggested that m6A methylation may participate in the pathological process of macrophages in syphilis.

### YTHDF1 Regulates Macrophage Polarization and the Inflammatory Response When Exposed to TP

**Figure 2:**
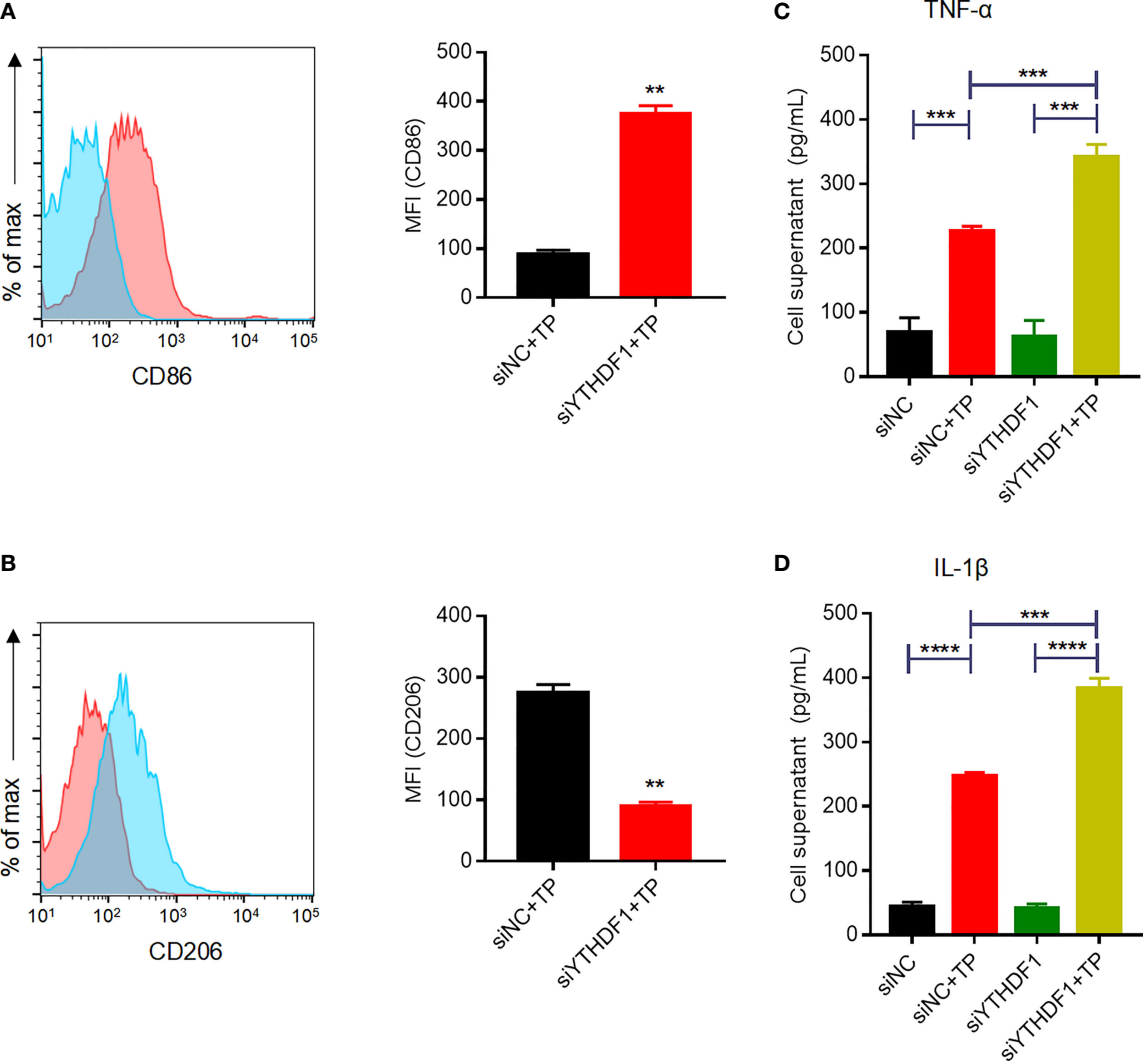
YTHDF1 regulates M2 macrophage polarization and suppresses the secretion of inflammatory cytokines in THP-1 cells infected with TP. **(A, B)** Flow cytometry profile of the expression of M1 and M2 markers (CD86 and CD206) in THP-1 cells infected with TP. **(C-D)** The concentrations of inflammatory cytokines (TNF-α and IL-1β) in the supernatants of THP-1 cells treated with TP. Data are shown as the mean ± S.D. **P < 0.01, ***P < 0.001,****P < 0.0001.

Given the upregulation of YTHDF1 protein and RNA in TP-infected macrophages, we next verified its role in macrophage polarization. YTHDF1 silencing mediated by siRNA transfection markedly inhibited at least 80% of the YTHDF1 protein expression (**Supplementary Figure S2**). Flow cytometry showed that YTHDF1 knockdown also upregulated the M1 markers (CD86 and iNOS) and downregulated the expression of M2 markers (CD206 and ARG1) (**Figures 2A, B** and **Supplementary Figure S3**), which demonstrated that YTHDF1 inhibited M1 polarization and induced M2 polarization. Moreover, TP infection significantly upregulated the secretion of inflammatory cytokines (TNF-α and IL-1β) by macrophages, which was further enhanced by YTHDF1 knockdown (**Figures 2C, D**).

### YTHDF1 and METTL3 Mediate the Protein Expression of SOCS3 in an m6A-Dependent Manner

**Figure 3:**
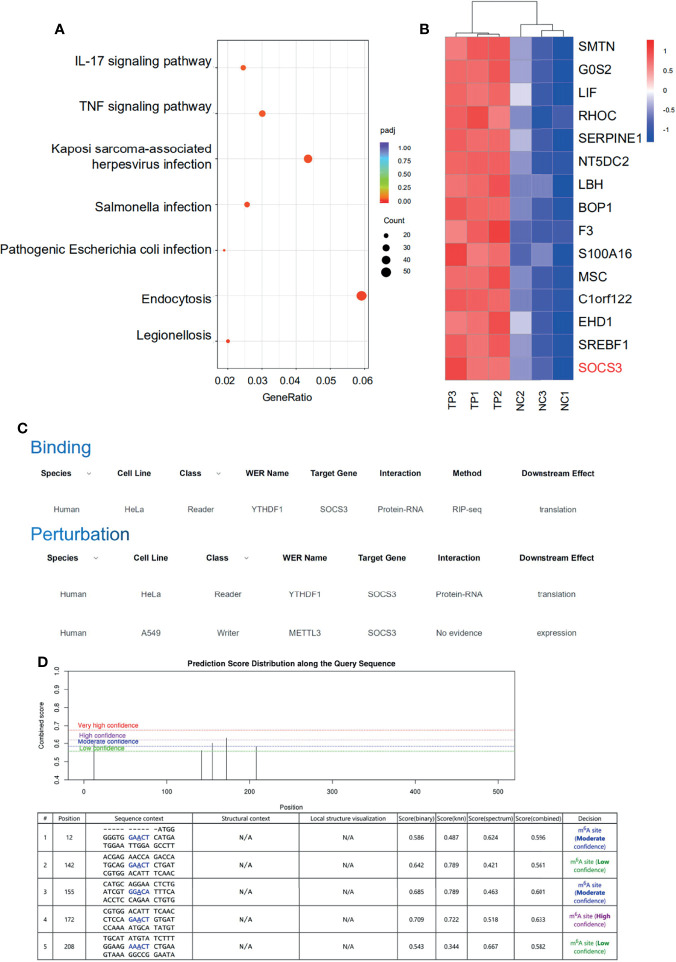
SOCS3 may be the target of the m6A reader YTHDF1 in TP-infected macrophages. **(A)** KEGG pathway analysis of biological processes associated with inflammation and infection in the RNA-seq dataset. **(B)** Heat map of the top 15 significantly upregulated mRNAs in TP-infected macrophages compared with control cells. **(C)** The association of binding and perturbation between YTHDF1 and SOCS3 was predicted by m6A2Target. **(D)** The potential m6A sites on SOCS3 mRNA were analyzed by m6Avar.

The RNA-sequencing results clearly showed that the mRNA expression patterns between the control and TP-infected macrophages significantly differed. Analysis of mRNA expression profiles showed that 3,110 genes were significantly dysregulated (log2-fold change > 0, P ≤ 0.05) in the TP-infected cells (859 upregulated genes and 1,251 downregulated genes) compared with those of the control group (**Supplementary Figure S4**). Kyoto Encyclopedia of Genes and Genomes analysis revealed that the upregulated genes in TP-infected macrophages were enriched in cytokine production and inflammatory signaling pathways such as the TNF signaling pathway, along with pathways associated with infection, and endocytosis (**Figure 3A**). We also investigated the top 15 upregulated genes (**Figure 3B**), which suggested that *SOCS3* may be associated with the negative regulation of the inflammatory response during TP infection. Furthermore, we predicted the association of YTHDF1 and SOCS3 with binding and perturbation using the m6A2Target tool^1^ (**Figure 3C**). Given that YTHDF1 functions by binding to m6A-methylated mRNA to promote its translation (9, 14), the potential m6A sites on *SOCS3* mRNA were analyzed using m6Avar, demonstrating high confidence for position 172 of *SOCS3* mRNA as an m6A site (**Figure 3D**).

**Figure 4:**
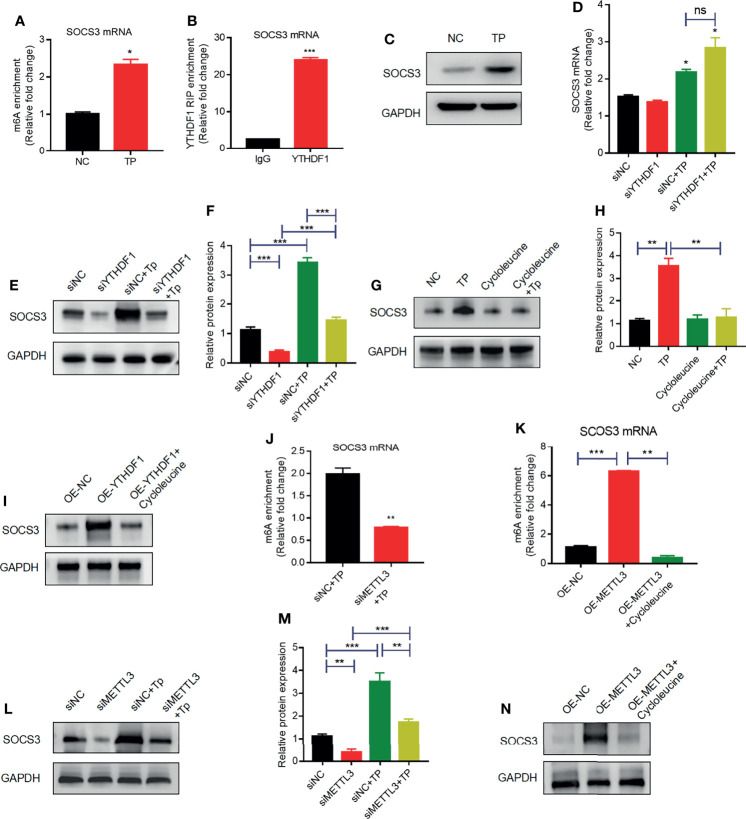
YTHDF1 and METTL3 regulate SOCS3 translation in an m6A-dependent manner. **(A)** MeRIP-qPCR analysis of the m6A levels of SOCS3 mRNA in THP-1 cells with or without TP infection. Total RNA was isolated from the NC group or TP-infected group. Methylated RNA was immunoprecipitated and then RT-qPCR analysis of the immunoprecipitated RNA was performed. **(B)** RIP analysis of the interaction of SOCS3 mRNA with YTHDF1 protein in THP-1 cells with or without TP infection. Enrichment of SOCS3 with YTHDF1 was assessed by RT-PCR and normalized to the input. **(C)** Western blot analysis of SOCS3 in THP-1 cells with or without TP infection. GAPDH was used as a control. **(D)** RT-PCR analysis of SOCS3 mRNA in THP-1 cells with or without TP infection following transfection with siNC or siYTHDF1. *P < 0.05 compared with siNC group. **(E, F)** Western blot analysis of SOCS3 in THP-1 cells with or without TP infection following transfection with siNC or siYTHDF1. GAPDH was used as a control. **(G, H)** Western blot analysis of SOCS3 in THP-1 cells with or without TP infection and with or without cycloleucine treatment. **(I)** Western blot analysis of SOCS3 in THP-1 cells transfected with control or YTHDF1 plasmid. **(J)** MeRIP-qPCR analysis of the m6A levels of SOCS3 mRNA in TP infected-THP-1 cells with or without METTL3 knockdown. **(K)** MeRIP-qPCR analysis of the m6A levels of SOCS3 mRNA in THP-1 cells transfected with control or YTHDF1 plasmid. **(L, M)** Western blot analysis of SOCS3 in THP-1 cells with or without TP infection following transfection with control or METTL3 knockdown. **(N)** Western blot analysis of SOCS3 in THP-1 cells transfected with control or METTL3 plasmid. *P < 0.05, **P < 0.01, ***P < 0.001; ns, no significance.

Indeed, the results of MeRIP-qPCR confirmed that TP infection increased m6A levels in *SOCS3* mRNA (**Figure 4A**). Furthermore, RIP-qPCR analysis revealed that *SOCS3* mRNA is a target of YTHDF1 protein in TP-infected THP-1 cells (**Figure 4B**). To ascertain whether *SOCS3* mRNA is a substrate for YTHDF1, we further examined the influence of YTHDF1 on the translation of SOCS3. Western blot analysis demonstrated that the SOCS3 expression level was elevated in the THP-1 cells after stimulation with TP (**Figure 4C**). YTHDF1 knockdown did not affect *SOCS3* mRNA expression (**Figure 4D**); however, knockdown of YTHDF1 significantly decreased the protein expression of SOCS3 (**Figures 4E, F**). Treatment with cycloleucine, an m6A methylation inhibitor, negatively regulated SOCS3 expression in the cells (**Figures 4G, H**). Furthermore, SOSC3 protein levels were elevated in THP-1 cells with YTHDF1 overexpression compared to those in control cells but were reduced after cycloleucine pretreatment (**Figure 4I**). In addition, we investigated whether METTL3 affects the m6A modification of SOCS3 mRNA. Western blotting showed that siRNA effectively repressed the expression of METTL3 (**Supplementary Figure S5**). Further MeRIP-qPCR analysis showed that, compared with that in the control group, the m6A modification level of *SOCS3* mRNA was decreased in the METTL3-knockdown group (**Figure 4J**). Besides, the m6A levels in the *SOCS3* mRNA were elevated in the THP-1 cells with METTL3 overexpression compared to those in control cells but were reduced after cycloleucine pretreatment (**Figure 4K**). Silencing *METTL3* impaired the protein expression of SOCS3 (**Figures 4L, M**). Protein levels of SOSC3 were elevated in the THP-1 cells with METTL3 overexpression compared to those in control cells but were reduced after cycloleucine pretreatment (**Figure 4N**). Collectively, these results indicate that METTL3 promotes the m6A modification of *SOCS3* mRNA, and that YTHDF1 recognizes the m6A-modified *SOCS3* mRNA to promote its translation.

### SOCS3 Negatively Regulates the Secretion of Cytokines by Macrophages Upon TP Infection *via* JAK2/STAT3 Signaling

**Figure 5:**
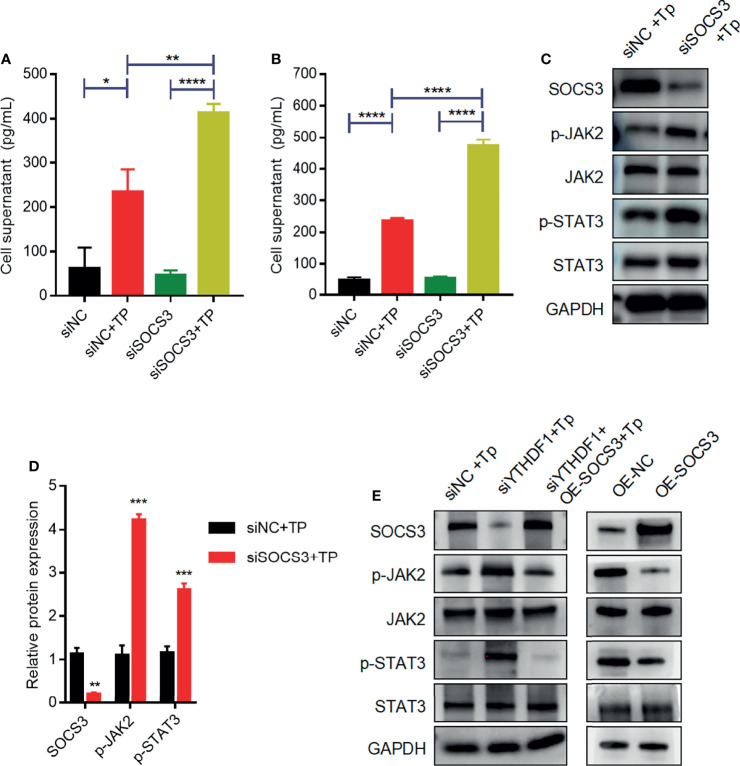
SOCS3 suppresses inflammation in TP-infected macrophages *via* the JAK2-STAT3 pathway. **(A, B)** The concentrations of inflammatory cytokines (TNF-α and IL-1β) in the supernatants of THP-1 cells with or without TP infection following transfection with siNC or siSOCS3. **(C, D)** Western blot analysis of SOCS3, p-JAK2, JAK2, p-STAT3, and STAT3 in THP-1 cells transfected with siNC or siSOCS3. **(E)** Western blot analysis of SOCS3, p-JAK2, JAK2, p-STAT3, and STAT3 in piPSCs with or without YTHDF1 knockdown and transfected with control or SOCS3 plasmid. *P < 0.05, **P < 0.01, ***P < 0.001, ****P < 0.0001.

Previous studies have demonstrated that SOCS3 is an important negative regulator of JAK2–STAT3 signaling, which plays an indispensable role in regulating the inflammatory response (9, 14). Based on the above findings, we investigated whether SOCS3 negatively affects the inflammatory response by inhibiting JAK2–STAT3 signaling in macrophages infected with TP. Compared with those of control cells, the secretion levels of inflammatory cytokines (TNF-α and IL-1β) increased in SOCS3-knockdown cells infected with TP (**Figures 5A, B**). Furthermore, silencing of *SOCS3* promoted the activation of JAK2–STAT3 signaling (**Figures 5C, D**). The overexpression of SOCS3 partially rescued the activation of JAK2–STAT3 signaling caused by YTHDF1 knockdown (**Figure 5E**).

## Discussion

Syphilis is a chronic, sexually transmitted disease that seriously endangers human health. The clinical manifestations of syphilis are extremely complex and involve almost all organs and systems in the body. In the early stages, syphilis infects the skin and mucosal membranes, and in the later stages, it causes irreversible damage to vital organs, including the nerves, bones, and cardiovascular system (15). Effective control and prevention of syphilis have become a serious social and public health problem of global concern (16). Studying the immune mechanisms of the human body after TP infection may help to find a strategy to eliminate syphilis.

When microorganisms invade, rapid cytokine production in the host is beneficial for their elimination; however, an exaggerated inflammatory response may cause severe systemic inflammation and organ dysfunction, such as the persistent inflammatory factor storm that occurs in patients severely infected with SARS-CoV-2 (17, 18). Understanding how the body controls the intensity of the secretion of inflammatory factors is a key focus of scientists studying microbial infections (19). As the main effectors of innate immunity, macrophages play an essential role in phagocytosis and clearance during microbial infection. Xu et al. (20) reported that there is a large amount of mononuclear macrophage infiltration in the skin lesions of patients with syphilis and rabbit models, and the level of the macrophage activator IFN-γ was significantly increased, along with the upregulation of the macrophage activation markers CD68, CD80, and CD86 in secondary syphilitic lesions (21). Previous studies have also shown that macrophages secrete IL-1β and promote TP phagocytosis *via* P2X7R (3). In addition, macrophages upregulate IL-6 and IL-8 expression *via* the TLR5 and MAPK/NF-κB signaling pathways when infected with TP (3). Thus, it has been confirmed that TP and its proteins induce an inflammatory response in macrophages. However, the specific negative intracellular regulators mediating these effects have not yet been thoroughly investigated. The negative effects of lipopolysaccharide (LPS), a gram-negative bacterial endotoxin that causes severe systemic inflammation and elicits numerous negative regulatory mechanisms, such as those involving A20, IRAKM, MyD88s, and SOCS1, have been investigated in a range of different hosts (19, 22). We speculate that many negative regulatory mechanisms participate in the inflammatory response caused by TP infection and the prevention of severe inflammation.

Increasing evidence has shown that m6A modification is related not only to normal physiological processes but also to immune processes involved when the body encounters pathogens. During viral and bacterial infection, m6A modifications regulate the inflammatory response and metabolic processes (23, 24). Xu et al. (25) demonstrated that LPS can significantly upregulate the expression and biological activity of METTL3 in macrophages, which induces inflammation *via* NF-κB. Additionally, they showed that the overexpression of METTL3 significantly inhibited the proliferation and reduced the inflammation in macrophages (25). Many other infection mechanisms that involve in m6A modification have been explored, such as those involving *Salmonella typhimurium* (23), vesicular stomatitis virus (24), and the gut microbiota (26). In this study, we provide the first evidence that the m6A modification level was enhanced in macrophages *in vitro* after TP infection and that the writer, METTL3, and the reader, YTHDF1, were upregulated both *in vitro* and in secondary syphilitic lesions. We inferred that METTL3 and YTHDF1 might be involved in the pathological processes of TP-stimulated macrophages. Indeed, YTHDF1 silencing experiments showed that YTHDF1 could inhibit the M1 polarization of macrophages and secretion of inflammatory factors, suggesting that YTHDF1 may be one of the key factors negatively regulating the inflammatory response of macrophages.

The SOCS family is typically induced during inflammation and infection, which stimulates the expression of the members and negatively regulates cytokine production through various signaling pathways (27, 28). SOCS3 is a negative regulator of the JAK-STAT pathway, which plays an important negative regulatory role in inflammatory diseases and cellular immune responses (29). The effect of SOCS3 on the immune response is achieved by inhibiting the signal transduction of LPS, IL-6, and other related mediators. The SH2 domain of SOCS3 competitively binds to JAK2 and blocks the activation of STAT3, thereby inhibiting the inflammatory factors, including STAT3 and TNF-α (29). Previous studies have shown that SOCS3 has a negative regulatory effect on autoimmune inflammation in macrophages and that the absence of SOCS3 can cause subacute inflammation in mice (30). In addition, SOCS3 is considered an important mediator of gastrointestinal inflammation, and the knocking out of SOCS3 in gastric epithelial cells can promote the occurrence of gastritis (31). In a mouse model of colitis, the methyltransferase METTL3 was shown to modify the mRNA levels of *Socs1* and *Socs3* mRNAs by m6A methylation, which reduced the attenuation of both mRNAs and increased their translation, thereby negatively regulating IL-7/STAT5 signaling and the inflammatory response in T cells (32). In this study, we found that m6A modification regulates SOCS3 translation in a METTL3/YTHDF1-orchestrated manner when macrophages are stimulated by TP. Mechanistically, we propose that METTL3 promotes the m6A methylation of *SOCS3* mRNA, and then YTHDF1 recognizes and binds to the m6A-containing mRNA of *SOCS3* and promotes SOCS3 protein expression. Previous studies have demonstrated that the JAK2–STAT3 pathway is involved in the expression and secretion of the inflammatory cytokines TNF-α and IL-1β (33, 34). Our results further suggest that YTHDF1 and SOCS3 negatively regulate the inflammatory response by regulating the JAK2–STAT3 pathway.

**Figure 6:**
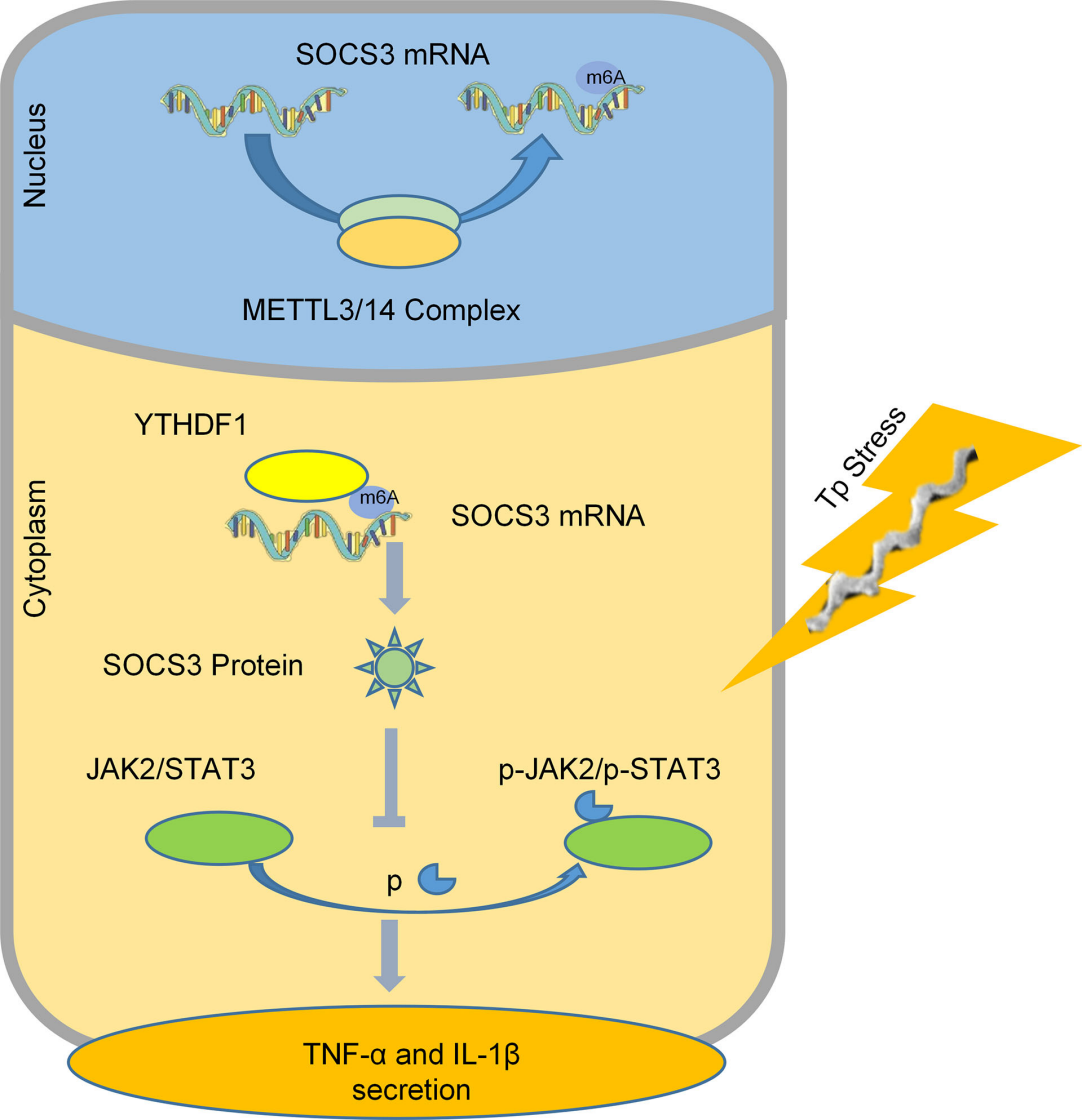
A schematic diagram revealing the proposed mechanism by which YTHDF1 mediates the protein expression of SOCS3 in an m6A-dependent manner and regulates the inflammatory response in macrophages infected with TP.

In summary, we found that after macrophages are stimulated by TP, YTHDF1, an m6A methylation reader is upregulated and it recognizes and binds to the m6A methylation site of *SOCS3* to promote its translation. SOCS3 inhibits the JAK2/STAT3 pathway and reduces the secretion of inflammatory factors, which achieves an anti-inflammatory effect (**Figure 6**). This study helps to explain why TP infection does not cause an excessive outburst of inflammation. Moreover, this study is the first to focus on the impact of m6A methylation in the pathological process of syphilis, providing new insights for exploring the mechanism of immune damage along with associated prevention and control strategies for syphilis, and improving our understanding of the pathogenesis of TP infection.

## Data Availability Statement

The datasets presented in this study can be found in online repositories. The names of the repository/repositories and accession number(s) can be found below: https://www.ncbi.nlm.nih.gov/, https://www.ncbi.nlm.nih.gov/bioproject/?term=PRJNA787979.

## Ethics Statement

The studies involving human participants were reviewed and approved by The Ethical Approval Board of Dermatology Hospital, Southern Medical University. The patients/participants provided their written informed consent to participate in this study.

## Author Contributions

ZL and MT: Experiments, Data curation, Writing - original draft. YJ, LZ, and XL: Preparation of TP. YL and BY: Supervision, Project administration, Writing - review and editing. All authors contributed to the article and approved the submitted version.

## Funding

This work was supported by the National Natural Science Foundation (NSFC) 81772240.

## Conflict of Interest

The authors declare that the research was conducted in the absence of any commercial or financial relationships that could be construed as a potential conflict of interest.

## Publisher’s Note

All claims expressed in this article are solely those of the authors and do not necessarily represent those of their affiliated organizations, or those of the publisher, the editors and the reviewers. Any product that may be evaluated in this article, or claim that may be made by its manufacturer, is not guaranteed or endorsed by the publisher.

## Footnotes

1
http://m6a2target.canceromics.org/

## References

[1] Keuning MW Kamp GA Schonenberg-Meinema D Dorigo-Zetsma JW van Zuiden JM Pajkrt D. Congenital Syphilis, the Great Imitator-Case Report and Review. Lancet Infect Dis (2020) 20:e173–9. doi: 10.1016/S1473-3099(20)30268-1 32502432

[2] Xu M Xie Y Jiang C Xiao Y Kuang X Wen Y. Treponema Pallidum Flagellins Elicit Proinflammatory Cytokines From Human Monocytes *via* TLR5 Signaling Pathway. Immunobiology (2017) 222:709–18. doi: 10.1016/j.imbio.2017.01.002 28126263

[3] Xu SL Lin Y Liu W Zhu XZ Liu D Tong ML. The P2X7 Receptor Mediates NLRP3-Dependent IL-1beta Secretion and Promotes Phagocytosis in the Macrophage Response to Treponema Pallidum. Int Immunopharmacol (2020) 82:106344. doi: 10.1016/j.intimp.2020.106344 32151957

[4] Lin LR Xiao Y Liu W Chen YY Zhu XZ Gao ZX. Development of Tissue Inflammation Accompanied by NLRP3 Inflammasome Activation in Rabbits Infected With Treponema Pallidum Strain Nichols. BMC Infect Dis (2018) 18:101. doi: 10.1186/s12879-018-2993-0 29490620 PMC5831842

[5] Carlson JA Dabiri G Cribier B Sell S. The Immunopathobiology of Syphilis: The Manifestations and Course of Syphilis Are Determined by the Level of Delayed-Type Hypersensitivity. Am J Dermatopathol (2011) 33:433–60. doi: 10.1097/DAD.0b013e3181e8b587 PMC3690623 21694502

[6] Sell S Norris SJ. The Biology, Pathology, and Immunology of Syphilis. Int Rev Exp Pathol (1983) 24:203–76. 6840996

[7] Huang T Yang J Zhang J Ke W Zou F Wan C. MicroRNA-101-3p Downregulates TLR2 Expression, Leading to Reduction in Cytokine Production by Treponema Pallidum-Stimulated Macrophages. J Invest Dermatol (2020) 140:1566–75. doi: 10.1016/j.jid.2019.12.012 31930972

[8] Han Z Wang X Xu Z Cao Y Gong R Yu Y. ALKBH5 Regulates Cardiomyocyte Proliferation and Heart Regeneration by Demethylating the mRNA of YTHDF1. Theranostics (2021) 11:3000–16. doi: 10.7150/thno.47354 PMC7806463 33456585

[9] Hu L Wang J Huang H Yu Y Ding J Yu Y. YTHDF1 Regulates Pulmonary Hypertension Through Translational Control of MAGED1. Am J Respir Crit Care Med (2021) 203:1158–72. doi: 10.1164/rccm.202009-3419OC 33465322

[10] Xu Z Peng B Cai Y Wu G Huang J Gao M. N6-Methyladenosine RNA Modification in Cancer Therapeutic Resistance: Current Status and Perspectives. Biochem Pharmacol (2020) 182:114258. doi: 10.1016/j.bcp.2020.114258 33017575

[11] Zhao BS Roundtree IA He C. Post-Transcriptional Gene Regulation by mRNA Modifications. Nat Rev Mol Cell Biol (2017) 18:31–42. doi: 10.1038/nrm.2016.132 27808276 PMC5167638

[12] Huang H Weng H Sun W Qin X Shi H Wu H. Recognition of RNA N(6)-Methyladenosine by IGF2BP Proteins Enhances mRNA Stability and Translation. Nat Cell Biol (2018) 20:285–95. doi: 10.1038/s41556-018-0045-z PMC5826585 29476152

[13] Liu T Wei Q Jin J Luo Q Liu Y Yang Y. The M6a Reader YTHDF1 Promotes Ovarian Cancer Progression *via* Augmenting EIF3C Translation. Nucleic Acids Res (2020) 48:3816–31. doi: 10.1093/nar/gkaa048 PMC7144925 31996915

[14] Zong X Xiao X Shen B Jiang Q Wang H Lu Z. The N6-Methyladenosine RNA-Binding Protein YTHDF1 Modulates the Translation of TRAF6 to Mediate the Intestinal Immune Response. Nucleic Acids Res (2021) 49:5537–52. doi: 10.1093/nar/gkab343 PMC8191762 33999206

[15] Hook ER. Syphilis. Lancet (2017) 389:1550–7. doi: 10.1016/S0140-6736(16)32411-4 27993382

[16] Peeling RW Mabey D Kamb ML Chen XS Radolf JD Benzaken AS. Syphilis. Nat Rev Dis Primers (2017) 3:17073. doi: 10.1038/nrdp.2017.73 29022569 PMC5809176

[17] Fu Y Cheng Y Wu Y. Understanding SARS-CoV-2-Mediated Inflammatory Responses: From Mechanisms to Potential Therapeutic Tools. Virol Sin (2020) 35:266–71. doi: 10.1007/s12250-020-00207-4 PMC7090474 32125642

[18] Channappanavar R Fehr AR Vijay R Mack M Zhao J Meyerholz DK. Dysregulated Type I Interferon and Inflammatory Monocyte-Macrophage Responses Cause Lethal Pneumonia in SARS-CoV-Infected Mice. Cell Host Microbe (2016) 19:181–93. doi: 10.1016/j.chom.2016.01.007 PMC4752723 26867177

[19] Du J Liao W Liu W Deb DK He L Hsu PJ. N(6)-Adenosine Methylation of Socs1 mRNA Is Required to Sustain the Negative Feedback Control of Macrophage Activation. Dev Cell (2020) 55:737–53. doi: 10.1016/j.devcel.2020.10.023 PMC7755741 33220174

[20] Lafond RE Lukehart SA. Biological Basis for Syphilis. Clin Microbiol Rev (2006) 19:29–49. doi: 10.1128/CMR.19.1.29-49.2006 16418521 PMC1360276

[21] Cruz AR Ramirez LG Zuluaga AV Pillay A Abreu C Valencia CA. Immune Evasion and Recognition of the Syphilis Spirochete in Blood and Skin of Secondary Syphilis Patients: Two Immunologically Distinct Compartments. PloS Negl Trop Dis (2012) 6:e1717. doi: 10.1371/journal.pntd.0001717 22816000 PMC3398964

[22] Kinjyo I Hanada T Inagaki-Ohara K Mori H Aki D Ohishi M. SOCS1/JAB Is a Negative Regulator of LPS-Induced Macrophage Activation. Immunity (2002) 17:583–91. doi: 10.1016/s1074-7613(02)00446-6 12433365

[23] Wu C Chen W He J Jin S Liu Y Yi Y. Interplay of m(6)A and H3K27 Trimethylation Restrains Inflammation During Bacterial Infection. Sci Adv (2020) 6:a647. doi: 10.1126/sciadv.aba0647 PMC7438091 32875102

[24] Liu Y You Y Lu Z Yang J Li P Liu L. N (6)-Methyladenosine RNA Modification-Mediated Cellular Metabolism Rewiring Inhibits Viral Replication. Science (2019) 365:1171–6. doi: 10.1126/science.aax4468 31439758

[25] Wang J Yan S Lu H Wang S Xu D. METTL3 Attenuates LPS-Induced Inflammatory Response in Macrophages *via* NF-kappaB Signaling Pathway. Mediators Inflamm (2019) 2019:3120391. doi: 10.1155/2019/3120391 31772500 PMC6854952

[26] Wang X Li Y Chen W Shi H Eren AM Morozov A. Transcriptome-Wide Reprogramming of N(6)-Methyladenosine Modification by the Mouse Microbiome. Cell Res (2019) 29:167–70. doi: 10.1038/s41422-018-0127-2 PMC6355850 30559439

[27] Klepsch O Namer LS Kohler N Kaempfer R Dittrich A Schaper F. Intragenic Regulation of SOCS3 Isoforms. Cell Commun Signal (2019) 17:70. doi: 10.1186/s12964-019-0379-6 31238931 PMC6593527

[28] Gao Y Zhao H Wang P Wang J Zou L. The Roles of SOCS3 and STAT3 in Bacterial Infection and Inflammatory Diseases. Scand J Immunol (2018) 88:e12727. doi: 10.1111/sji.12727 30341772

[29] Durham GA Williams J Nasim MT Palmer TM. Targeting SOCS Proteins to Control JAK-STAT Signalling in Disease. Trends Pharmacol Sci (2019) 40:298–308. doi: 10.1016/j.tips.2019.03.001 30948191

[30] Zhang X Wang Y Yuan J Li N Pei S Xu J. Macrophage/microglial Ezh2 Facilitates Autoimmune Inflammation Through Inhibition of Socs3. J Exp Med (2018) 215:1365–82. doi: 10.1084/jem.20171417 PMC5940261 29626115

[31] Zhang H Wang Y Li S Tang X Liang R Yang X. SOCS3 Protects Against Neonatal Necrotizing Enterocolitis *via* Suppressing NLRP3 and AIM2 Inflammasome Activation and P65 Nuclear Translocation. Mol Immunol (2020) 122:21–7. doi: 10.1016/j.molimm.2020.03.019 32278838

[32] Li HB Tong J Zhu S Batista PJ Duffy EE Zhao J. m(6)A mRNA Methylation Controls T Cell Homeostasis by Targeting the IL-7/STAT5/SOCS Pathways. Nature (2017) 548:338–42. doi: 10.1038/nature23450 PMC5729908 28792938

[33] Li Q Cheng Y Zhang S Sun X Wu J. TRPV4-Induced Müller Cell Gliosis and TNF-α Elevation-Mediated Retinal Ganglion Cell Apoptosis in Glaucomatous Rats *via* JAK2/STAT3/NF-κB Pathway. J Neuroinflamm (2021) 18:271. doi: 10.1186/s12974-021-02315-8 PMC8596927 34789280

[34] Yin L Dai Q Jiang P Zhu L Dai H Yao Z. Manganese Exposure Facilitates Microglial JAK2-STAT3 Signaling and Consequent Secretion of TNF-A and IL-1β to Promote Neuronal Death. Neurotoxicology (2018) 64:195–203. doi: 10.1016/j.neuro.2017.04.001 28385490

