# Supplementary material for: YTHDF1 Negatively Regulates Treponema pallidum-Induced Inflammation in THP-1 Macrophages by Promoting SOCS3 Translation in an m6A-Dependent Manner
Source: Front Immunol. 2022 Apr 4;13:857727. doi: 10.3389/fimmu.2022.857727 (PMC9013966; doi:10.3389/fimmu.2022.857727)
Supplement: Supplementary file 1 [file DataSheet_1.docx]

Supplementary Material

[Supplymentary Figure 1. qPCR analysis of YTHDF1 mRNA in THP-1 cells with or without TP infection. GAPDH was used as an internal control.***P < 0.001. 1](#_Toc1057)

[Supplymentary Figure 2. YTHDF1 knockdown efficiency was measured by western blot. siYTHDF1 -1 was selected for subsequent experiments. 2](#_Toc29326)

[Supplymentary Figure 3. A-B RT-PCR analysis of CD86, CD206, iNOS and ARG1 mRNA in THP-1 cells with or without TP infection and with or without YTHDF1 knowdown. C. Western blot analysis of iNOS and ARG1 in THP-1 cells with or without TP infection and with or without YTHDF1 knowdown.*P < 0.05, **P < 0.01, ***P < 0.001, ****P < 0.0001. 3](#_Toc5277)

[Supplymentary Figure 4. The volcano plot of the RNA-seq dataset of macropahged infected by TP. 4](#_Toc26713)

[Supplymentary Figure 5. METTL3 knockdown efficiency was measured by western blot. siMETTL3 -3 was selected for subsequent experiments. 5](#_Toc31131)

[SupplymentaryTable1. siRNA sequences for target genes. 6](#_Toc12773)


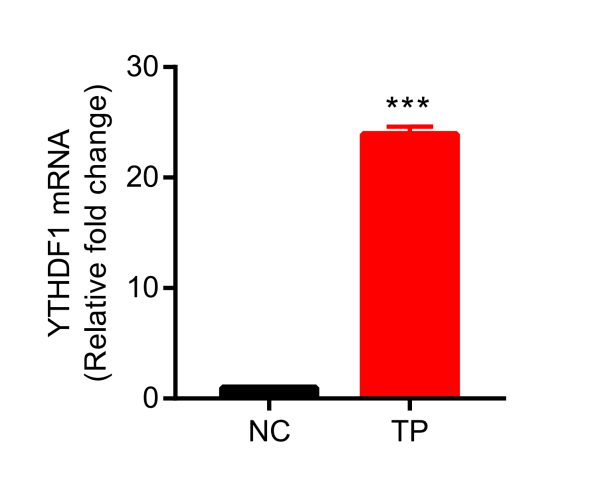


Supplymentary Figure 1. qPCR analysis of YTHDF1 mRNA in THP-1 cells with or without TP infection. GAPDH was used as an internal control.***P < 0.001.


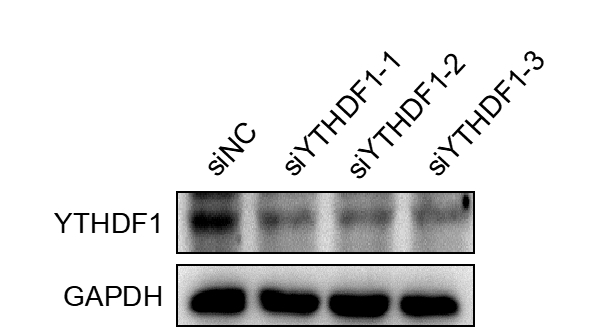


Supplymentary Figure 2. YTHDF1 knockdown efficiency was measured by western blot. siYTHDF1 -1 was selected for subsequent experiments.


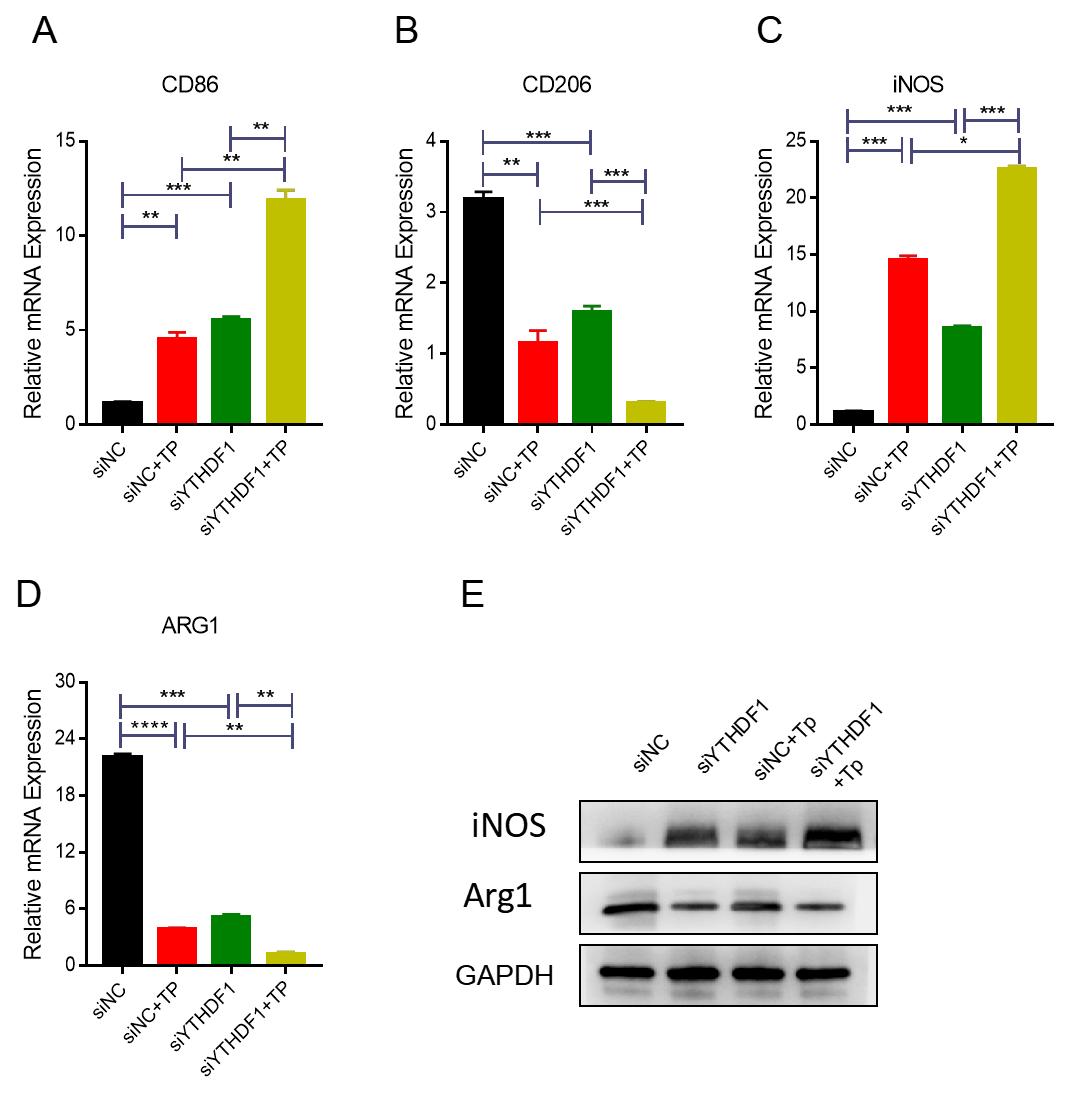


Supplymentary Figure 3. **(A-D)**. RT-PCR analysis of CD86, CD206, iNOS and ARG1 mRNA in THP-1 cells with or without TP infection and with or without YTHDF1 knowdown. **(E)**. Western blot analysis of iNOS and ARG1 in THP-1 cells with or without TP infection and with or without YTHDF1 knowdown.*P < 0.05, **P < 0.01, ***P < 0.001, ****P < 0.0001.


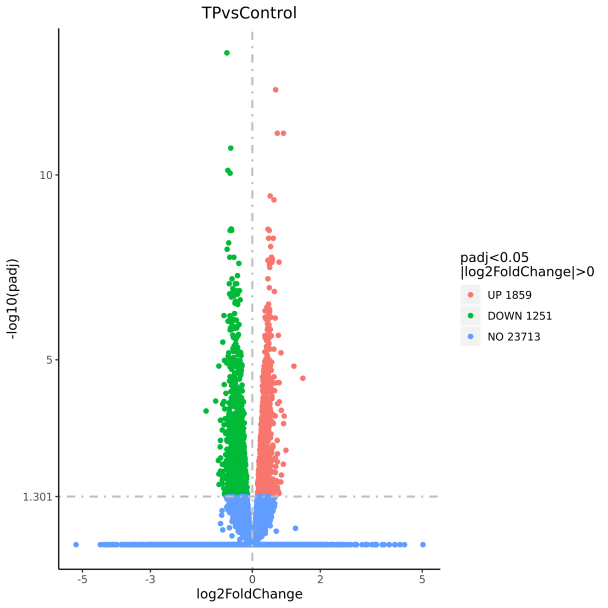


TP VS Control

-log10(padj)

log2FoldChange

Supplymentary Figure 4. The volcano plot of the RNA-seq dataset of macropahged infected by TP.


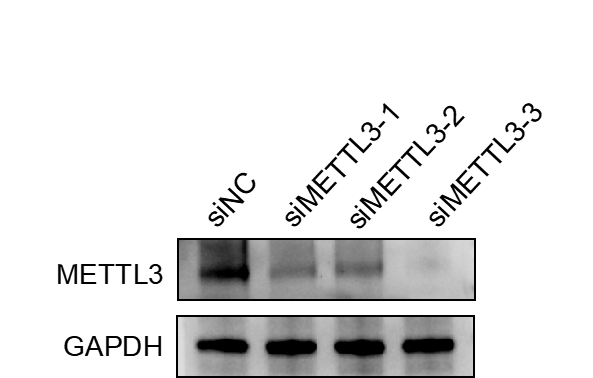


Supplymentary Figure 5. METTL3 knockdown efficiency was measured by western blot. siMETTL3 -3 was selected for subsequent experiments.

SupplymentaryTable1. siRNA sequences for target genes.

| Target genes | siRNA sequences | |
| --- | --- | --- |
|  | sense（5'-3'） | antisense（5'-3'） |
| YTHDF1 | CCCGAAAGAGUUUGAGUGGAATT | UUCCACUCAAACUCUUUCGGGTT |
| METTL3 | GCAAGAAUUCUGUGACUAUTT | GCUAACACCCGUACUATT |
| SOCS3 | CCA AGA ACC UGC GCA UCC AdTdT | UGG AUG CGC AGG UUC UUG GdTdT |
